# Survival following relapse in childhood haematological malignancies diagnosed in 1974–2003 in Yorkshire, UK

**DOI:** 10.1038/sj.bjc.6603667

**Published:** 2007-03-06

**Authors:** R G Feltbower, S E Kinsey, M Richards, G Shenton, M P Michelagnoli, P A McKinney

**Affiliations:** 1 Paediatric Epidemiology Group, Centre for Epidemiology and Biostatistics, 30–32 Hyde Terrace, University of Leeds, Leeds LS2 9LN, UK; 2 Paediatric Oncology & Haematology, Leeds Teaching Hospitals NHS Trust, Beckett St, Leeds LS9 7TF, UK; 3 University College Hospital, 235 Euston Road, London, NW1 2BU, UK

**Keywords:** relapse, risk factors, survival, children

## Abstract

We examined population-based information on relapsed childhood haematological cancers, investigating factors that might influence both overall survival and survival following relapse among the 1177 children (0–14 years) diagnosed with a haematological malignancy in Yorkshire from 1974 to 2003, of whom 342 (29%) relapsed at least once. Leukaemia patients from more deprived areas were significantly less likely to relapse (odds ratio=0.54, 95% confidence interval 0.32–0.93 for most deprived quintile *vs* least deprived quintile; *P*_trend_=0.06), especially those with acute myeloid leukaemia (*P*=0.04). Neither ethnic group nor distance to the main treatment centre was associated with risk of relapse. Overall, patients who relapsed at least once had 5-year survival rates of 46% (41–51%) compared with 79% (76–81%) of those who did not. Five-year survival rates from the time of first relapse increased from 20% in 1974–1983 to 45% in 1984–2003. Length of first remission was a strong predictor of survival for leukaemia with a 46% reduced risk of death for every additional year of event-free survival. Of children who experienced a relapse, 46% survived at least 5 years, whereas just under half of patients survived 5 years beyond disease recurrence. This provides a baseline for future comparisons and demonstrates that relapsed childhood cancer need not imply a poor outcome.

Population based survival rates now exceed 70% for all malignancies diagnosed under the age of 15 in the UK (Coleman *et al*, 1999), having improved steadily since 1950 (Stiller and Bunch, 1990). Despite improved prognosis, an appreciable number of patients still fail first-line treatment, with 75% of deaths occurring in paediatric oncology units (Al-Asiri *et al*, 1992) and among long-term survivors of childhood cancer in a population-based series (Robertson *et al*, 1994) being due to recurrent disease.

In an audit of care carried out in the 1990s in a tertiary paediatric oncology unit in Leeds (unpublished), relapsed disease accounted for up to 50% of all inpatient bed occupancy, constituting a major part of the unit's workload. Most literature on relapse concerns acute lymphoblastic leukaemia (ALL), and is almost entirely limited to patients either on a trial protocol or selected from a hospital-based series (Eden *et al*, 2000; Gustafsson *et al*, 2000; Lawson *et al*, 2000; Schrappe *et al*, 2000; Silverman *et al*, 2000; Tsuchida *et al*, 2000; Vilmer *et al*, 2000; Chessells *et al*, 2003; Roy *et al*, 2005), although one paper from Scandinavia has reported on the outcome following relapse ALL in a population-based cohort (Schroeder *et al*, 1995). A smaller number of studies have focused on relapse associated with acute myeloid leukaemia (Caspers and Creutzig, 2005; Gibson *et al*, 2005; Lie *et al*, 2005).

We examined population-based information on relapsed childhood haematological cancers, looking at factors that might influence both overall survival and survival following relapse; we also tested the local clinical impression that time to relapse was increasing in response to more intensive treatment.

## MATERIALS AND METHODS

Diagnostic, clinical and demographic information on all children diagnosed under the age of 15 with a haematological malignancy from 1974 to 2003 was obtained from the Yorkshire Specialist Register of Cancer in Children and Young People, covering the former Yorkshire Regional Health Authority (Feltbower *et al*, 2001). Checks with adjoining childhood cancer registries and the National Registry of Childhood Tumours (Stiller *et al*, 1998) are carried out routinely to ensure optimum ascertainment rates. Overall, 90% of diagnoses were confirmed by histopathology, although this proportion increased substantially over time, averaging 98% since 1986. Children were actively followed up every 2 years with general practitioners and hospital records to determine their health status, treatment and any adverse outcomes such as relapse or second malignancies. Our clinical definition of ‘relapse’ was recurrent disease, either occurring locally at the same site as the initial diagnosis and/or elsewhere, and the date of this event was recorded. A cross-check with the national clinical trials database (www.ctsu.ox.ac.uk) was undertaken to ensure all recurrent episodes were recorded and trial entry status was complete.

Each patient's diagnosis was coded with ICD-O-2/ICD-10 morphology and site codes and categorised according to the International Classification of Childhood Cancer (ICCC) as either leukaemia (group I) or lymphoma (group II) (Kramárová *et al*, 1996). Information on the following explanatory risk factors was derived for each patient: deprivation, ethnicity and distance from main treating centre. The full address and postcode of each patient at diagnosis was validated and linked to a small census area (enumeration district, ED). The Townsend deprivation score was assigned to each patient based on the 1991 census, including unemployment, car ownership, housing tenure and household overcrowding (Townsend *et al*, 1988) and categorised into quintiles. The Townsend index has been shown to be a reliable measure of an individual's social class (Danesh *et al*, 1999). Distance to treatment centre of more or less than 20 km from the main treating centre was derived by allocating a 100 m grid reference to the location of each subject's residence (by ED centroid) together with their treating hospital within the Yorkshire Region. Ethnic group, defined as south Asian or not, was assigned to each individual based on their full name according to a south Asian names data dictionary (Nam Pehchan, City of Bradford Metropolitan District Council, 2002) and verified using a secondary source (London School of Hygiene and Tropical Medicine, 1999). The Nam Pehchan software has been shown to be a robust measure of categorising ethnic origin (Cummins *et al*, 1999).

The overall risk of a first relapse episode occurring was analysed using logistic regression in relation to the following factors: age at diagnosis, sex, period of diagnosis, deprivation, ethnic group, entry into a randomised clinical trial and distance from residence to the main hospital of treatment. We excluded from the regression modelling any patients who died before reaching remission to ensure that the nonrelapse comparison group was homogeneous. The remission period was defined as 5 weeks postdiagnosis following standard evaluation of patient response to induction therapy at this time (Gaynon *et al*, 1997) and because most patients will have achieved remission within this period. To compare the risk for those who relapsed *vs* those who never relapsed, we report odds ratios (OR) and 95% confidence intervals (CI). Each model's goodness-of-fit was assessed (Hosmer and Lemeshow, 1989). For patients who had relapsed at least once, Cox regression was used to assess whether the risk of death was influenced by time to first relapse, the variables listed above or the site of first relapse (bone marrow±other sites (BM), central nervous system (CNS), testis and other). All patients were followed up until 31 May 2006.

## RESULTS

Hosmer–Lemeshow goodness-of-fit tests showed that all the models fitted the data well.

### Relapse patterns

One thousand one hundred and seventy-seven children were identified, 856 with leukaemia and 321 with lymphoma. Three hundred and forty-two (29%) of these patients relapsed at least once (Table 1). Of the 18 patients diagnosed with Burkitt lymphoma, only two relapsed, so it was decided not to combine non-Hodgkin's lymphoma (NHL; ICCC group IIb) and Burkitt lymphoma (ICCC group IIc) together in the statistical analysis.

Children with lymphoma were significantly less likely to relapse (20%) than those with leukaemia (32%): OR=0.53 (95% CI 0.39–0.72). Median time to relapse showed some differences: patients with ALL and Hodgkin's lymphoma relapsed after 2.2 years (range 1 month to 18 years), whereas those with acute myeloid leukaemia (AML), ‘other’ leukaemia (ICCC groups Ic–Ie), NHL and ‘other’ lymphoma (ICCC groups IIc–IIe) relapsed after 0.7–1.5 years (range 1 month to 24 years). There was no variation in time to first relapse by diagnostic period.

### Factors associated with relapsed disease

Of the 200 individuals who died but never relapsed, 77 did so within 5 weeks of their diagnosis date and were excluded from the logistic regression modelling: these mainly consisted of ALL (*n*=27), AML (*n*=26) and NHL (*n*=15).

### Leukaemia

Females with ALL were significantly less likely to relapse than males (OR=0.69; 95% CI 0.51–0.95), with AML displaying a similar but nonsignificant association (OR=0.43; 95% CI 0.18–1.01). Older children (ages 10–14) with leukaemia had a slightly higher risk of relapse compared with 0–4 year old children (OR=1.41; 95% CI 0.94–2.11), although this effect was not significant for any diagnostic subgroup.

Children diagnosed with leukaemia in 1984–1993 and 1994–2003 were significantly less likely to relapse than those diagnosed in 1974–1983 (*P*_trend_=0.01). This effect was not entirely explained by reduced rates of early relapse: for example, the proportion of children who relapsed 3 years postdiagnosis decreased over time from 16% in 1974–1983 to 6% in 1994–2003. Patients entered into a clinical trial were significantly less likely to relapse than those who were not.

Children in the most deprived areas had a significantly lower risk of relapse than those from the most affluent areas (*P*_trend_=0.06; OR=0.54, 95% CI 0.32–0.93 for most deprived quintile *vs* least deprived quintile). This effect was most marked for those with AML (*P*_trend_=0.04). No significant differences in risk of relapse were evident according to ethnic group or distance to the main treatment centre.

### Lymphoma

No significant differences by gender were observed for lymphoma. The general pattern of a lower risk of relapse for those diagnosed more recently was observed, especially for NHL, although no significant trends were found. Trial entry was associated with a significant reduction in risk of relapse, although no consistent associations were seen with deprivation. Neither ethnic group nor distance to treatment centre influenced the risk of relapse.

### Factors associated with survival following relapse

**Figure 1:**
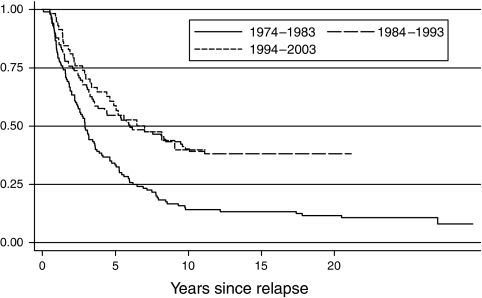
Postrelapse survival trends by period of diagnosis for childhood leukaemia diagnosed between 1974 and 2003 in Yorkshire, UK.

Median survival times for leukaemia and lymphoma following the first relapse were 1.5 and 1.15 years, respectively. Overall, patients who relapsed at least once had 5-year survival rates from diagnosis of 46% (95% CI 41–51%) compared with 79% (76–81%) for those who never relapsed. Results were similar for other diagnostic groups except AML, where 5-year survival rates were 30 and 46% for relapsed and nonrelapsed diseases, respectively. Survival rates for leukaemia from the time of first relapse (Figure 1) showed that only 20% of patients survived beyond 5 years if diagnosed in 1974–1983 compared with 45% if diagnosed in 1984–2003 (*P*_trend_=0.01). Patients with AML showed an even more dramatic improvement in 5-year survival rates, rising from 0% in 1974–1983 to 35% in 1984–1993 and 53% in 1994–2003.

Table 2 presents the hazard ratio (HR) estimates and 95% CI derived from the Cox regression analysis for the 342 patients who experienced at least one relapse episode. Results for ‘other’ leukaemia (ICCC groups Ic–e), Hodgkin's lymphoma (IIa), NHL (IIb) and ‘other’ lymphoma (IIc–e) are not shown because of small numbers.

### Leukaemia

Females had a nonsignificantly increased risk of death compared with males for leukaemia (HR=1.32, 95% CI 0.96–1.82) and all other diagnostic subgroups. A significantly increased risk of death was observed for 10–14-year-olds with leukaemia compared with those aged 0–4 years (HR=1.53, 95% CI 1.05–2.23).

For both ALL and AML, individuals diagnosed in the two most recent decades were significantly less likely to die than those diagnosed in 1974–1983 (*P*_trend_=0.001). No significant differences in survival were found by ethnic group, although patients treated on a trial protocol had a nonsignificantly reduced risk of death. Increasing levels of deprivation were associated with an increased risk of death for leukaemia, but no significant trends were observed for any subgroup. Time to first relapse was strongly associated with length of survival for all diagnostic subgroups: for all leukaemia combined, patients were 46% less likely to die for every additional year of event-free survival (HR=0.54, 95% CI 0.47–0.62). Site of relapse had a significant impact on survival for ALL: testis and CNS relapses had significantly higher survival rates compared with BM. Distance to the main treatment centre did not influence survival.

### Lymphoma

No significant difference in survival was observed for lymphoma patients by gender, age, period of diagnosis, ethnicity or distance to the main treatment centre. Trial entry did not show any beneficial effect on survival, revealing a slightly elevated risk of death. No significant trends across deprivation quintiles were observed, although individuals from the least deprived areas were least likely to die. Time to first relapse had an impact on survival, albeit less pronounced than that for leukaemia (HR=0.87, 95% CI 0.74–1.02). No significant differences in survival were observed by site of relapse.

## DISCUSSION

We report for the first time factors influencing the risk of relapsed childhood haematological cancer from a UK cohort spanning 30 years, together with their subsequent survival chances. Our findings indicate that 30% of leukaemia and 20% of lymphoma patients are likely to relapse at least once. Time to first relapse was similar for ALL and Hodgkin's lymphoma, although somewhat shorter for AML, other leukaemia subtypes and NHL, but accorded with previous ALL reports (Gustafsson *et al*, 1987, 2000; Eden *et al*, 2000; Lawson *et al*, 2000; Schrappe *et al*, 2000; Silverman *et al*, 2000; Tsuchida *et al*, 2000; Vilmer *et al*, 2000; Chessells *et al*, 2003; Roy *et al*, 2005). There was no change in the median time to relapse for any diagnostic group across the study period, which therefore did not confirm local impressions that more children were surviving longer at the expense of more frequent relapses.

A key finding was the 46% 5-year survival rates for those who had relapsed, in contrast to 79% for those who did not relapse. This observation was seen across all diagnostic groups except AML and is consistent with recent international and UK data on ALL (Schroeder *et al*, 1995; Chessells, 1998; Lawson *et al*, 2000; Chessells *et al*, 2003; Roy *et al*, 2005), although our population-based study, as well as other diagnostic groups, included a longer follow-up period. Furthermore, almost half (45%) of leukaemia and lymphoma patients diagnosed since 1984 survived 5 years after their initial relapse episode, a striking improvement from the one in five (20%) 5-year survivors diagnosed in 1974–1983. Recurrent disease therefore need not imply a poor outcome for childhood haematological cancer.

Time to first relapse was an important prognostic indicator, in agreement with other international and UK findings for ALL (Schroeder *et al*, 1995; Chessells, 1998; Lawson *et al*, 2000; Chessells *et al*, 2003; Roy *et al*, 2005), our data indicating a 46% improvement in survival for leukaemia for every year of event-free survival since diagnosis. Localisation of relapse was also a significant prognostic factor for ALL, with testis and CNS relapses exhibiting significantly better survival than BM relapse. Increased survival following relapse was observed for leukaemia patients entered into a clinical trial and for those diagnosed more recently. Five-year overall survival rates of 45% for patients who had relapsed in our Yorkshire cohort diagnosed in the 1990s is only marginally lower than data reported from UK ALL R1 and R2 trials showing 56% overall survival during the same period (Lawson *et al*, 2000; Roy *et al*, 2005). Equivalent postrelapse survival rates for AML showed a consistent improvement over the study period from 0% in 1974–1983 up to 53% in 1994–2003. This correlates well with a general trend in the reduction of the cumulative risk of relapse for patients on trials (Caspers and Creutzig, 2005; Lie *et al*, 2005) and agrees with 5-year post-relapse survival rates of around 30% reported from other UK data between 1988 and 1995 (Gibson *et al*, 2005). Females who had relapsed had a slightly (but not significantly) greater risk of death than males, in contrast to the usual finding that females survive longer than males for most diagnostic groups (Coleman *et al*, 1999); this requires confirmation in other populations.

Leukaemia patients from more deprived areas were significantly less likely to relapse initially than those from the most affluent areas, although this effect was largely restricted to AML. This novel finding is rather puzzling, as it is unlikely to be due to lack of access to treatment as most young children visit tertiary referral centres both for their main therapy and long-term follow-up; moreover, distance to treatment centre was not associated with risk of relapse. Nor could it be explained by any variation in fatality rate before reaching remission by level of deprivation. For those patients with complete stage of tumour recorded, more aggressive cancers were not related to deprivation category. Given the relatively small numbers who had relapsed with leukaemia, the associations with deprivation may be due to chance.

Females with leukaemia were less likely to relapse than males, as were younger patients, supporting previous UK findings for ALL (Lawson *et al*, 2000). Those diagnosed during the earliest decade (1974–1983) had the poorest prognosis irrespective of leukaemia subtype. NHL followed the same patterns as leukaemia in terms of the effect on relapse from sex and period of diagnosis, whereas Hodgkin's lymphoma exhibited a nonsignificantly higher risk for female patients, and only those diagnosed in the most recent decade (1994–2003) had a reduced risk of relapse compared to earlier registrations.

The statistical power of our analyses was dependent firstly on the number of relapses observed within each diagnostic group (eg 277 out of 856 for leukaemia) and secondly by the number of deaths experienced. This precluded more detailed investigations of relapse patterns and survival trends. Also, some patients diagnosed in the most recent period will have not experienced relapsed disease due to censoring, although analyses took this into account. Details of biological markers and molecular genetics were not uniformly available for the Yorkshire cohort. However, biological information can be a useful tool for indicating survival chances following relapse. For instance, for relapsed ALL, the presence of 11q23 abnormalities and Ph^1^-positive ALL may be linked with a poorer prognosis (Vora *et al*, 1996; Chessells *et al*, 2003) whereas survivors with the TEL-AML 1 fusion transcript had an extended second remission period compared with those without (Seeger *et al*, 1997). Differences in the molecular characteristics of tumours occurring across deprivation quintiles may partly explain some of our findings.

The observation that almost half of all patients with any haematological malignancy (except AML) registered since 1974 who fail first-line treatment and relapse survive 5 years beyond their diagnosis and the same proportion survive 5 years beyond their initial relapse episode is an important finding as it has been achieved without an increase in the number of long-term relapse episodes: it provides a baseline figure for future studies. Links between deprivation and the risk of relapse and subsequent survival require validation in other population-based cohorts.

## Figures and Tables

**Table 1:** Proportion of childhood (0–14 years) haematological patients having relapsed according to survival status, diagnosed between 1974–2003 in Yorkshire, UK

		**Patients alive at 31 May 2006**		**Deceased patients**		**All registered patients**		
**ICCC^a^**		**Ever relapsed**		**Ever relapsed**		**Ever relapsed**		
**group**	**Diagnostic group**	**Yes (%)**	**No (%)**	**Yes (%)**	**No (%)**	**Yes (%)**	**No (%)**	**Total**
I	Leukaemia	78 (15.2%)	435 (84.8%)	199 (58.0%)	144 (42.0%)	277 (32.4%)	579 (67.6%)	856
Ia	Acute lymphoid leukaemia	66 (15.0%)	375 (85.0%)	162 (69.8%)	70 (30.2%)	228 (33.9%)	445 (66.1%)	673
Ib	Acute myeloid leukaemia	12 (20.7%)	46 (79.3%)	31 (34.4%)	59 (65.6%)	43 (29.1%)	105 (70.9%)	148
Ic–e	Other leukaemia^b^	0 (0.0%)	14 (100.0%)	6 (28.6%)	15 (71.4%)	6 (17.1%)	29 (82.9%)	35
II	Lymphoma	27 (11.9%)	200 (88.1%)	38 (40.4%)	56 (59.6%)	65 (20.2%)	256 (79.8%)	321
IIa	Hodgkin's lymphoma	16 (13.3%)	104 (86.7%)	5 (33.3%)	10 (66.7%)	21 (15.6%)	114 (84.4%)	135
IIb	Non-Hodgkin's lymphoma	10 (13.5%)	64 (86.5%)	26 (43.3%)	34 (56.7%)	36 (26.9%)	98 (73.1%)	134
IIc–e	Other lymphoma^b^	1 (3.0%)	32 (97.0%)	7 (36.8%)	12 (63.2%)	8 (15.4%)	44 (84.6%)	52
	Total	105 (14.2%)	635 (85.8%)	237 (54.2%)	200 (45.8%)	342 (29.1%)	835 (70.9%)	1177

a ICCC: International Classification of Childhood Cancer (Kramárová *et al*, 1996).

b Excluded from main analyses.

**Table 2:** Cox regression: association between risk factors and survival following relapsed childhood haematological cancer diagnosed between 1974–2003 in Yorkshire, UK

	**Leukaemia (ICCC Ia**–**e)**		**ALL (ICCC^a^ Ia)**		**AML (ICCC^a^ Ib)**		**Lymphoma (ICCC^a^ IIa**–**e)**	
**Factor**	**HR**	**95% CI**	**HR**	**95% CI**	**HR**	**95% CI**	**HR**	**95% CI**
*Sex*								
Males	1.00		1.00		1.00		1.00	
Females	1.32	0.96–1.82	1.20	0.82–1.75	2.06	0.65–6.55	1.06	0.43–2.59
								
*Age group*								
0–4	1.00		1.00		1.00		1.00	
5–9	0.98	0.69–1.40	0.88	0.59–1.30	3.16	0.46–21.37	1.48	0.50–4.39
10–14	1.53	1.05–2.23	1.33	0.86–1.07	2.59	0.84–7.99	1.22	0.41–3.61
								
*Period* *of diagnosis*								
1974–1983	1.00		1.00		1.00		1.00	
1984–1993	0.55	0.38–0.79	0.59	0.39–0.88	0.95	0.26–3.51	1.36	0.45–4.10
1994–2003	0.39	0.26–0.61	0.43	0.27–0.69	0.19	0.04–0.84	2.28	0.71–7.29
								
*Entered clinical trial*								
No	1.00		1.00		1.00		1.00	
Yes	0.74	0.52–1.07	0.77	0.49–1.22	1.76	0.40–7.67	1.16	0.49–2.73
								
*Ethnic group*								
Non-south Asian	1.00		1.00		1.00		1.00	
South Asian	0.85	0.45–1.62	1.06	0.49–2.29	0.22	0.02–2.87	1.20	0.36–3.95
								
*Deprivation*								
I (lowest)	1.00		1.00		1.00		1.00	
II	1.26	0.79–2.00	1.43	0.86–2.36	0.51	0.09–2.99	4.87	1.18–20.06
III	0.97	0.61–1.54	0.87	0.52–1.48	2.48	0.45–13.71	3.98	1.06–14.92
IV	1.25	0.79–1.98	0.99	0.59–1.66	1.68	0.33–8.54	1.48	0.44–5.01
V (highest)	1.71	1.03–2.84	1.62	0.92–2.86	0.46	0.07–3.01	2.15	0.59–7.78
	*P*_trend_=0.11		*P*_trend_=0.57		*P*_trend_=0.92		*P*_trend_=0.50	
Time to 1st relapse (years)	0.54	0.47–0.62	0.54	0.46–0.64	0.47	0.23–0.97	0.87	0.74–1.02
								
*Distance to treatment hospital*: <20 km	1.00		1.00		1.00		1.00	
⩾20 km	1.17	0.84–1.64	1.04	0.71–1.53	1.07	0.33–3.45	1.28	0.53–3.06
								
*Site of relapse*								
Bone marrow	1.00		1.00		1.00		1.00	
Testis	0.43	0.24–0.75	0.35	0.19–0.63	—	—	0.91	0.09–9.59
Central Nervous system	0.42	0.24–0.72	0.50	0.28–0.89	—	—	0.35	0.06–2.03
Other	0.86	0.50–1.48	0.79	0.43–1.44	0.62	0.06–6.02	0.60	0.22–1.65

ALL: acute lymphoblastic leukaemia; AML: acute myeloid leukaemia; CI, confidence interval; HR, hazard ratio;

a ICCC: International Classification of Childhood Cancer (Kramárová *et al*, 1996)
